# Capturing Successful Aging in Daily Life: Exploring the Intensive Longitudinal Findings From a U.S. National Sample

**DOI:** 10.1093/geront/gnae121

**Published:** 2024-09-02

**Authors:** Dwight C K Tse, Kelsey P Finley, Linzi F Crawford, Jeanne Nakamura

**Affiliations:** Department of Psychological Sciences and Health, University of Strathclyde, Glasgow, UK; Quality of Life Research Center, Claremont Graduate University, Claremont, California, USA; Department of Psychological Sciences and Health, University of Strathclyde, Glasgow, UK; Quality of Life Research Center, Claremont Graduate University, Claremont, California, USA

**Keywords:** Daily diary, Intraindividual variability, Multilevel modeling

## Abstract

**Background and Objectives:**

Thus far, successful aging has been conceptualized and operationalized as a relatively static construct. Investigating daily successful aging provides a dynamic approach highlighting how successful aging can be achieved through everyday actions, giving older adults a stronger sense of control over their lives.

**Research Design and Methods:**

We used 8-day diary data from Midlife in the United States 2’s U.S. national sample of older adults (*N* = 716, *M*_age_[standard deviation, *SD*] = 68.80[6.45]). Participants reported daily physical symptoms, functioning, and engagement in life (collectively, successful aging indicators), alongside daily stressors and positive events. We also correlated the personal mean and standard deviation of the indicators with 1-time measures of health and well-being.

**Results:**

Intraclass correlation revealed substantial within-person variability in successful aging indicators. These indicators were associated with daily stressors and positive events. One-time health and well-being indicators were positively associated with personal mean levels of successful aging, although their correlations with personal *SD*s were less consistent.

**Discussion and Implications:**

Intraindividual variations in successful aging as everyday symptoms, functioning, and engagement with life are observable among a national sample of older adults, challenging the static view of successful aging and, instead, emphasizing the need to understand “micro-level” contributors of successful aging.

Successful aging has been defined as distinct from normative or usual aging to inspire research on dimensions and predictors of aging well (Rowe & Kahn, 1987, 1997; Schaie, 2016). Although there is little consensus on the definition of successful aging, most researchers agree that it is a multidimensional construct (Martin et al., 2015; Settersten, 1999). Among other successful aging models (for reviews, see Depp & Jeste, 2006; Martinson & Berridge, 2015; Teater & Chonody, 2020), the prevailing one proposed by Rowe and Kahn (1997, 2015) defines successful aging with three dimensions “encompassing the avoidance of disease and disability, the maintenance of high physical and cognitive function, and sustained engagement in social and productive activities” (1997, p. 433).

Despite its widespread use, Rowe and Kahn’s model also faces scrutiny for potentially excluding those with normative age-related health conditions (e.g., Calasanti & King, 2021; Strawbridge et al., 2002). Many older adults with existing health conditions or disability have demonstrated good cognitive function and overall well-being (Carpentieri et al., 2017), and have been shown to rate themselves as aging successfully (Romo et al., 2013). Strawbridge et al. (2002) propose potential unintended consequences of the model such as celebrating the genetically fortunate few, placing blame on the less fortunate majority, and discouraging those who are classified as aging “unsuccessfully” from pursuing secondary and tertiary prevention. Kahn (2002) responds by stating that he is sympathetic to the concern, although he believes this unintended consequence of dichotomous thinking “reflects a characteristic of contemporary American culture rather than something intrinsic to the concept” (p. 726). We suggest that the successful aging model may find new and unique applications by employing intensive longitudinal methods such as a daily diary design.

To provide an example from related work, the study of subjective views of aging has benefitted from utilizing intensive longitudinal methods. This research has shown that older adults’ views on aging and awareness of age-related changes are subject to proximal factors and changes in daily contexts (e.g., Bellingtier et al., 2017; Hoffman et al., 2022). In line with these findings, Cohn-Schwartz and Gerstorf (2022) underscore the usefulness of intensive longitudinal methods to study specific everyday life experiences that make people feel younger or older. In relation to successful aging, the “missing voice” perspective, labeled in Martinson and Berridge’s (2015) review, posits that any successful aging model should consider older adults’ subjective views, as opposed to relying solely on established objective criteria, putting greater emphasis on older adults’ subjective perceptions of aging well (Pruchno et al., 2023; Shrira et al., 2016). A phenomenological, dynamic measure of successful aging could integrate more subjectivity by moving down to the experiential level while still retaining the three dimensions of the Rowe and Kahn model. Therefore, such a measure of successful aging has the potential to make a contribution distinct from global subjective perceptions of aging well, if it shows variation at the day level and is sensitive to day-to-day conditions.

A glimpse into the assessment of within-person variation of specific successful aging dimensions and related constructs suggests that we might expect daily variation and that this kind of assessment lends itself to a novel understanding of aging well. Daily/momentary assessment of social engagement, a dimension of successful aging, has proven to have unique contributions to our understanding of influences on well-being in older adults through contextual moderators that are only feasible to assess using intensive longitudinal designs. For example, Mann et al. (2022) found that older adults’ engagement in prosocial activity attenuates the negative relationship between solitude (i.e., absence of social interactions) and momentary well-being. Even dimensions of successful aging that appear to be relatively stable and perhaps contribute to its needless exclusivity, like avoidance of disease and disability, have shown variations at a more micro time scale. For example, for people with rheumatoid arthritis, daily variations in subjective pain were associated with the disease, and these daily variations were predicted by depression, happiness, and frustration (Schneider et al., 2012). Rather than designating someone with arthritis as not aging successfully, examination of the day-to-day variation in pain would allow for the identification of ideal days, environments, and structures that promote the experience of successful aging within this population and others potentially excluded from successful aging research.

Using intensive longitudinal methods would allow researchers to get closer to the lived experience of successful aging and investigate its proximal facilitators and barriers that are otherwise obscured by solely using global one-time measures (Palmier-Claus et al., 2019). As such, successful aging becomes less exclusive but retains its usefulness—allowing researchers to examine proximal predictors of successful aging, including the successful management of symptoms associated with disease and disability. It may prove useful to conceptualize and measure successful aging as a dynamic condition varying within-person across time distinct from a static status or characteristic. However, for day-to-day assessments of successful aging to be utilized in the ways we have proposed, we first must confirm that successful aging does, in fact, vary within-person (see Hypothesis 1).

If successful aging indeed varies within-person, a follow-up question will be whether there are proximal factors that are associated with its day-to-day variations. Cross-sectionally, stressful events have been found to be negatively related to successful aging (e.g., Byun & Jung, 2016; Chukwuorji et al., 2017), whereas social support (e.g., Gow et al., 2007; Moore et al., 2015) has been conceptualized as both predictors (positive) of successful aging and moderators (attenuators) of the relationship between health factors and successful aging. Using intensive longitudinal methods, Graf et al. (2017) found that intraindividual variation in daily hassle and daily uplift intensity was negatively and positively related, respectively, to intraindividual changes in self-assessed health—one dimension of Rowe and Kahn’s (1997) successful aging construct. However, to our knowledge, there is no study examining daily predictors of *intraindividual* variation on a *multidimensional* measure of successful aging. If there is substantial within-person variability in successful aging, it may be responsive to within-person variability in daily encounters such as those discussed above (see Hypothesis 2).

The evaluation of daily successful aging should also consider its association with crucial life outcomes in late adulthood. Drawing from the literature, one-time measures of successful aging tend to be related to health and well-being. In Depp and Jeste’s (2006) review, successful aging was strongly associated with better health, such as the absence of hearing problems, fewer impairments in activities of daily living, and being a nonsmoker. For engagement with life, Sabia et al. (2012) found that healthy behaviors, such as diet and physical activity, which are more disposed to change than general health factors, were related to successful aging in a follow-up survey more than a decade later. Cho et al. (2015) found that dimensions of successful aging such as cognitive functioning and (the absence of) physical impairment were associated with better subjective well-being. Strawbridge et al. (2002) found positive associations between both a self-rated and a criterion-based measure of successful aging and numerous measures of well-being. Windsor et al. (2015) found that a higher sense of purpose was related to indicators of aging well. Taken together, we expect that daily assessments of successful aging will also be related to global assessments of health and well-being, similar to their associations with one-time successful aging measures depicted in the literature (see Hypothesis 3).

## The Current Study

Analyzing secondary data from a diary study with a large sample of older adults, we investigated successful aging in terms of daily functioning and experiences rather than one-off measurements. Providing a dynamic approach and investigating day-to-day predictors may introduce a more achievable perspective of successful aging for most older adults. This is important as it may provide older adults with higher levels of perceived control over their lives and aging processes, as well as more positive self-perceptions of aging (Kotter-Grühn & Hess, 2012). Both have been shown to be associated with better functioning and well-being (Levy et al., 2002).

H1. Daily successful aging indicators will show considerable within-person variations.

H2. Daily successful aging indicators are responsive to day-to-day events.

H3. Daily successful aging indicators will correlate with one-time measures of health and well-being.

## Research Design and Methods

### Data and Sample

Participants were from the Midlife in the United States (MIDUS) 2 Daily Diary Project that included a national sample of U.S. Americans (Radler, 2014). Data were collected in 2004–2009 via 8 consecutive days of surveys and phone interviews. Additionally, we combined the participants’ data with that from one-time interviews and surveys conducted as part of the MIDUS 2 project and the MIDUS 2 Cognitive Functioning project. Excluding participants who were aged below 60 during the diary project (*n* = 1,226) and those who had not completed all the components (*n* = 80), the final sample included 716 participants (6,368 total daily reports) with age ranging from 60 to 84, *M*_age_(standard deviation, *SD*) = 68.80(6.45), 57.3% female, 92.5% White.

### Measures

#### Within-person variables

##### Daily successful aging

Successful aging indicators were extracted from daily survey items that matched with Rowe and Kahn’s (1997) conceptualization of successful aging, including daily physical symptoms (e.g., “backache”; 30 symptoms in total), subjective physical (“active”) and cognitive functioning (“attentive”), social connectedness (“like you belong,” “close to others”), and perceived productivity (“proud,” “enthusiastic,” “confident”). Except for daily physical symptoms, participants responded to the items with the prompt “how much of the time today did you feel ...” on a 5-point scale ranging from 0 (*None of the time*) to 4 (*All of the time*). Daily physical symptoms were recoded to 0–4+, given the distribution and the need to transform it to a 5-point scale. We computed the daily successful aging composite by reversing the physical symptom score, taking the average of the items within the subcomponents, and averaging the scores across components. Higher scores reflect a higher degree of daily successful aging.

When examining the reliability of these items, we conducted multilevel confirmatory factor analysis using R packages lavaan and semTools to account for the multilevel data and second-order factor structure (Jorgensen et al., 2022; Rosseel, 2012). The model showed a good fit with the data, compartive fit index (CFI) = 0.964, root mean square error of approximation (RMSEA) = 0.043, 90% CI [0.038, 0.048], standardized root mean square residual (SRMR)_within_ = 0.022, SRMR_between_ = 0.038. The between- and within-person reliability, sometimes referred to as McDonald’s ω (Jorgensen et al., 2022), was 0.91 and 0.69, respectively, indicating that the scale had satisfactory internal consistency at both levels. We also examined the between-person reliability (using personal means; see Analytic methods) using more familiar statistics. Cronbach’s alpha for the personal means was 0.82, with item-total correlations ranging from 0.33 (daily symptoms) to 0.82 (daily productivity).

##### Daily stressors and positive events

Participants selected from a list of 7 potential stressors (e.g., interpersonal conflict) and 5 potential positive events (e.g., sharing a good laugh) the ones they experienced on each day (Almeida et al., 2002). The total numbers of reported daily stressors and positive events were added up to create summative composite scores of daily stressors and positive events, respectively. Because very rarely did participants report more than 3 stressors or positive events, we recoded both composites to a 4-point scale (0, 1, 2, 3+).

#### Between-person variables

##### One-time successful aging measures

From MIDUS 2 one-time surveys/interviews, we extracted survey items that corresponded to the daily successful aging indicators, where participants were asked with what frequency they felt “active,” “attentive,” “like you belong,” “close to others,” “proud,” “enthusiastic,” and “confident,” each measured on a 5-point scale ranging from 0 (*None of the time*) to 4 (*All of the time*). We also recoded the 30-item physical symptoms checklist in a 5-point format with 0–4+ symptoms. Similar to the daily counterpart, we computed a one-time successful aging composite by taking the average of the items within the subcomponents, and averaging the scores across components (Cronbach’s α = 0.90).

##### Health and well-being

We included six one-time indicators of physical health (instrumental activities of daily living [IADLs]; chronic health conditions, self-evaluated physical health, health compared to others your age, vigorous activity, and moderate activity), five indicators of cognitive functioning (Brief Test of Adult Cognition by Telephone [BTACT], episodic memory, executive functioning, memory compared to others your age, self-perception of intellectual abilities), and five indicators of mental well-being (satisfaction with life, Ryff’s psychological well-being, social well-being, self-evaluated mental/emotional health, and perceived control) in the between-person analyses. See Supplementary Material for the descriptions of these variables.

#### Analytic methods

We first estimated multilevel null models with daily successful aging indicators as the outcome variables. Intraclass correlations (ICCs) obtained from these models reflected the variation accounted for at both between- and within-person levels (H1). As ICCs are calculated by dividing between-person variance by the total variance (sum of between- and within-person variance), lower ICC values represent larger proportions of variance accounted for by within-person variations instead of between-person differences. Then, we included in the models the numbers of daily stressors and positive events as predictors (H2). The predictors were separated into the personal mean and the person-mean centered component to estimate simultaneously their between- and within-person effects on daily successful aging (Curran & Bauer, 2011).

For between-level analyses, we computed the personal means (*M*_Personal_) of daily successful aging indicators by taking the personal averages across the 8 days. We correlated these means with the one-time version of the same items to examine their construct validity. We also correlated *M*_Personal_ of daily successful aging indicators with the one-time indicators of health and well-being (H3). Additionally, we explored the zero-order and partial correlations between daily successful aging personal standard deviation (*SD*_Personal_) and one-time health and well-being indicators, given that within-person variance was a unique piece of information that one-time measurement of successful aging cannot capture. For analyses at both levels, we reported the findings with 1,000 bootstrapped samples to account for potential deviations from the parametric assumptions.

SPSS Syntax files are publicly available at https://osf.io/bc8hs/ (SPSS, Chicago, IL).

## Results

### Within-Person Analyses

#### Intraclass correlations

In the null models, the ICCs of the daily successful aging indicators, reflecting the proportion of variance accounted for by between-person differences (as opposed to within-person variation), ranged from 54.24% (physical functioning) to 72.47% (overall successful aging; see Table 1). In other words, more than a quarter of the variance of each indicator was accounted for by within-person, day-level variations, which was consistent with H1.

**Table 1.** Estimates (Bootstrapped 95% CIs) of Multilevel Models

Variable	Successful aging	Disease/disability	Physical functioning	Cognitive functioning	Social engagement	Productivity
Stressors_B_	−0.57^***^ [−0.70, −0.44]	0.79^***^ [0.54, 1.04]	−0.43^***^ [−0.60, −0.26]	−0.50^***^ [−0.65, −0.35]	−0.50^***^ [−0.65, −0.35]	−0.64^***^ [−0.80, −0.49]
Positive events_B_	0.19^***^ [0.12, 0.27]	−0.02 [−0.17, 0.13]	0.20^***^ [0.10, 0.30]	0.28^***^ [0.19, 0.37]	0.25^***^ [0.16, 0.34]	0.19^***^ [0.10, 0.29]
Stressors_W_	−0.09^***^ [−0.12, −0.07]	0.15^***^ [0.12, 0.20]	−0.06^**^ [−0.09, −0.02]	−0.07^***^ [−0.10, −0.04]	−0.07^***^ [−0.10, −0,04]	−0.08^***^ [−0.11, −0.06]
Positive events_W_	0.02 [−0.00, 0.03]	0.1^***^ [0.06, 0.14]	0.06^***^ [0.03, 0.08]	0.03^*^ [0.00, 0.05]	0.05^***^ [0.03, 0.08]	0.04^***^ [0.02, 0.06]
ICC_null_	0.724	0.631	0.542	0.560	0.642	0.707
PVR_L2_	0.081	0.052	0.037	0.072	0.056	0.078
PVR_L1_	0.075	0.055	0.038	0.042	0.064	0.057

*Notes*: *k* = 6,368; *N* = 716. ICC_null_ = intraclass correlation of the null model; PVR_L2_ and PVR_L1_ = proportion of variance reduced at level 2 and level 1, respectively. Subscripts B and W refer to between- and within-person components of the predictor, respectively.

^*^ *p* < .05.

^**^ *p* < .01.

^***^ *p* < .001.

#### Daily stressors and positive events

As shown in Table 1, at the within-person level, daily stressors and positive events were associated negatively and positively, respectively, with most successful aging indicators, although the effect of within-person daily positive events on overall daily successful aging was non-significant at *p* = .068. That is, on days when an older adult encountered more stressors than usual (i.e., higher than their average number), they reported lower levels of successful aging. We also found similar effects of between-person stressors and positive events on these indicators, such that when comparing two older adults, the one reporting fewer daily stressors and more positive events on average was more likely to score high in daily successful aging than the other. Surprisingly, however, on days with more-than-usual daily positive events, older adults also experienced more daily physical symptoms. Taken together, the findings regarding H2 were mixed: amid the highlighted exceptions, daily successful aging indicators were responsive to daily events mostly as anticipated.

### Between-Person Analyses

#### Convergence with one-time successful aging measures

The correlation matrix (Table 2) revealed that (a) *M*_Personal_ of daily successful aging indicators were positively associated with one-time successful aging measurements (for physical symptoms, the associations with other indicators were negative; 0.13<|*r*|s < 0.58), and (b) the correlations between each daily indicator’s *M*_Personal_ and their corresponding one-time measure (median *r* = 0.49) were generally larger than off-diagonal correlations with non-corresponding measures (median *r* = 0.39). This suggests satisfactory construct validity of the daily indicators.

**Table 2.** Correlation Coefficients and Bootstrapped 95% Confidence Intervals Between Personal Means (*M*_Personal_) of Successful Aging Indicators and the Corresponding One-Time Successful Aging Measures

Diary variable (*M*_Personal_)	MIDUS 2 one-time equivalence					
	SAG	DIS	PHY	COG	SOC	PRO
Successful aging (SAG)	**0.58** ^***^ **[0.52, 0.64]**	−0.38^***^ [−0.45, −0.31]	0.48^***^ [0.41, 0.54]	0.44^***^ [0.37, 0.51]	0.39^***^ [0.32, 0.46]	0.53^***^ [0.47, 0.59]
Disease/disability (DIS)	−0.33^***^ [−0.40, −0.27]	**0.47** ^***^ **[0.41, 0.54]**	−0.27^***^ [−0.34, −0.20]	−0.17^***^ [0.26, −0.10]	−0.13^**^ [−0.20, −0.05]	−0.24^***^ [−0.32, −0.17]
Physical functioning (PHY)	0.52^***^ [0.03, 0.57]	−0.26^***^ [0.33, −0.18]	**0.51** ^***^ **[0.44, 0.57]**	0.40^***^ [0.32, 0.46]	0.32^***^ [0.25, 0.39]	0.47^***^ [0.40, 0.53]
Cognitive functioning (COG)	0.50^***^ [0.43, 0.56]	−0.22^***^ [−0.29, −0.14]	0.40^***^ [0.32, 0.46]	**0.46** ^***^ **[0.40, 0.53]**	0.35^***^ [0.28, 0.42]	0.46^***^ [0.40, 0.53]
Social engagement (SOC)	0.47^***^ [0.40, 0.54]	−0.20^***^ [−0.28, −0.12]	0.33^***^ [0.25, 0.40]	0.37^***^ [0.29, 0.44]	**0.46** ^***^ **[0.38, 0.53]**	0.44^***^ [0.37, 0.50]
Productivity (PRO)	0.53^***^ [0.47, 0.59]	−0.25^***^ [−0.32, −0.17]	0.43^***^ [0.36, 0.49]	0.41^***^ [0.34, 0.48]	0.39^***^ [0.32, 0.46]	**0.55** ^***^ **[0.49, 0.61]**

*Notes*: *N* = 716. Correlation coefficients measuring the same construct (correlations at the diagonal) are bolded.

^**^ *p* < .01.

^***^ *p* < .001.

#### Personal mean of daily successful aging

Table 3 shows that the *M*_personal_ of daily successful aging was significantly correlated with one-time physical health (from *r* = −0.37 with chronic health conditions to *r* = 0.36 with IADL), cognitive functioning (from *r* = 0.09 with executive functioning and BTACT to *r* = 0.32 with memory compared with others), and mental well-being variables (from *r* = 0.20 with social well-being to *r* = 0.42 with satisfaction with life). That is, older adults who reported greater successful aging across the data collection period indeed enjoyed better physical health, cognitive functioning, and mental well-being, as assessed by the one-time measures. These were mostly consistent with H3, although the effect sizes ranged only between very small and moderate.

**Table 3.** Correlation Coefficients and Bootstrapped 95% Confidence Intervals Between Personal Means (*M*_Personal_) and Standard Deviations (*SD*_Personal_) of Successful Aging Indicators and One-Time Health and Well-Being Measures

Variables	Successful aging	Disease/disability	Physical functioning	Cognitive functioning	Social engagement	Productivity
	*M*_Personal_					
Physical health						
IADL	0.36** [0.30, 0.43]	−0.45** [−0.41, −0.39]	0.36** [0.29, 0.42]	0.16** [0.08, 0.23]	0.10** [0.03, 0.18]	0.21** [0.14, 0.28]
Chronic health conditions	−0.37** [−0.44, −0.29]	0.46** [0.40, 0.52]	−0.25** [−0.34, −0.18]	−0.18** [−0.26, −0.10]	−0.18** [−0.28, −0.09]	−0.23** [−0.32, −0.15]
Vigorous activity	0.11** [0.04, 0.19]	−0.10** [−0.17, −0.03]	0.15** [0.08, 0.22]	0.07 [−0.01, 0.14]	0.04 [−0.04, 0.11]	0.09* [0.01, 0.16]
Moderate activity	0.14** [0.06, 0.21]	−0.08* [−0.16, −0.01]	0.17* [0.10, 0.25]	0.11** [0.03, 0.19]	0.07 [−0.01, 0.15]	0.09* [0.01, 0.17]
Self-evaluated physical health	0.35** [0.29, 0.42]	−0.35** [−0.41, −0.28]	0.34** [0.27, 0.41]	0.22** [0.15, 0.30]	0.18** [0.10, 0.25]	0.23** [0.15, 0.30]
Health compared to others your age	0.31** [0.23, 0.37]	−0.26** [−0.33, −0.18]	0.32** [0.24, 0.40]	0.21** [0.13, 0.28]	0.17** [0.10, 0.26]	0.21** [0.13, 0.29]
Cognitive functioning						
BTACT composite	0.09* [0.02, 0.17]	−0.12** [−0.19, −0.04]	0.07 [0.00, 0.14]	0.09* [0.02, 0.17]	0.03 [−0.04, 0.11]	0.01 [−0.05, 0.08]
Episodic memory	0.10** [0.03, 0.17]	−0.07 [−0.14, 0.01]	0.10** [0.03, 0.17]	0.10** [0.02, 0.18]	0.06 [−0.02, 0.13]	0.07 [0.01, 0.13]
Executive functioning	0.09* [0.01, 0.16]	−0.13** [−0.20, −0.05]	0.04 [−0.02, 0.11]	0.08* [0.01, 0.16]	0.04 [−0.04, 0.11]	0.00 [−0.07, 0.08]
Memory compared to others your age	0.32** [0.25, 0.39]	−0.24** [−0.31, −0.16]	0.31** [0.22, 0.38]	0.26** [0.18, 0.33]	0.20** [0.11, 0.28]	0.26** [0.18, 0.34]
Personality in intellectual aging contexts	0.22** [0.00, 0.04]	−0.15** [−0.22, −0.07]	0.22** [0.14, 0.30]	0.19** [0.11, 0.27]	0.13** [0.05, 0.21]	0.18** [0.11, 0.26]
Mental well-being						
Satisfaction with life	0.42** [0.35, 0.48]	−0.29** [−0.36, −0.22]	0.35** [0.27, 0.42]	0.32** [0.24, 0.40]	0.36** [0.28, 0.43]	0.33** [0.26, 0.41]
Psychological well-being composite	0.40** [0.33, 0.47]	−0.15** [−0.22, −0.07]	0.37** [0.30, 0.44]	0.35** [0.28, 0.41]	0.40** [0.33, 0.47]	0.39** [0.32, 0.46]
Social well-being composite	0.20** [0.12, 0.27]	−0.06 [−0.14, 0.01]	0.19** [0.11, 0.27]	0.15** [0.07, 0.23]	0.23** [0.15, 0.31]	0.16** [0.08, 0.24]
Self-evaluated mental/emotional health	0.32** [0.25, 0.39]	−0.22** [−0.29, −0.15]	0.25** [0.17, 0.32]	0.26** [0.18, 0.32]	0.27** [0.20, 0.34]	0.28** [0.20, 0.35]
Perceived control	0.35** [0.28, 0.42]	−0.21** [−0.29, −0.13]	0.34** [0.27, 0.42]	0.25** [0.17, 0.32]	0.28** [0.19, 0.35]	0.31** [0.24, 0.39]
	*SD*_Personal_					
Physical health						
IADL	−0.21** [−0.28, −0.13]	−0.09* [−0.17, −0.00]	−0.22** [−0.30, −0.15]	−0.22** [−0.29, −0.14]	−0.16** [−0.24, −0.08]	−0.20** [−0.28, −0.13]
Chronic health conditions	0.18** [0.10, 0.27]	0.07 [−0.02, 0.17]	0.17** [0.10, 0.25]	0.17** [0.08, 0.26]	0.13** [0.04, 0.21]	0.18** [0.10, 0.25]
Vigorous activity	−0.11** [−0.18, −0.03]	−0.03 [−0.10, 0.06]	−0.13** [−0.20, −0.05]	−0.09* [−0.17, −0.02]	−0.11** [−0.19, −0.03]	−0.15** [−0.22, −0.07]
Moderate activity	−0.07 [−0.16, 0.01]	0.02 [−0.06, 0.10]	−0.10** [−0.18, −0.02]	−0.13** [−0.21, −0.06]	−0.08* [−0.16, 0.01]	−0.10* [−0.18, −0.02]
Self-evaluated physical health	−0.23** [−0.30, −0.15]	−0.10** [−0.18, −0.02]	−0.24** [−0.31, −0.16]	−0.23** [−0.30, −0.17]	−0.22** [−0.28, −0.14]	−0.19** [−0.25, −0.12]
Health compared to others your age	−0.17** [−0.24, −0.10]	−0.01 [−0.09, 0.07]	−0.18** [−0.26, −0.11]	−0.18** [−0.26, −0.10]	−0.17** [−0.24, −0.09]	−0.13** [−0.20, −0.05]
Cognitive functioning						
BTACT composite	−0.09* [−0.16, −0.01]	−0.07 [−0.14, 0.01]	−0.04 [−0.12, 0.03	−0.15** [−0.22, −0.08]	−0.11** [−0.19, −0.04]	−0.07 [−0.14, 0.01]
Episodic memory	−0.07 [−0.14, 0.01]	−0.02 [−0.19, 0.06]	−0.10* [−0.18, −0.02]	−0.13** [−0.19, −0.06]	−0.07 [−0.15, 0.02]	−0.07 [−0.14, 0.01]
Executive functioning	−0.08* [−0.15, −0.00]	−0.08 [−0.15, 0.00]	−0.01 [−0.09, 0.06]	−0.12** [−0.19, 0.04]	−0.11** [−0.18, −0.03]	−0.05 [−0.12, 0.03]
Memory compared to others your age	−0.20** [−0.28, −0.14]	−0.06 [−0.13, 0.03]	−0.22** [−0.29, −0.14]	−0.26** [−0.33, −0.19]	−0.23** [−0.31, −0.17]	−0.21** [−0.28, −0.14]
Personality in intellectual aging contexts	−0.09* [−0.17, −0.02]	0.02 [−0.05, 0.09]	−0.12** [−0.19, −0.04]	−0.15** [−0.22, −0.08]	−0.13** [−0.21, −0.06]	−0.15** [−0.23, −0.08]
Mental well-being						
Satisfaction with life	−0.21** [−0.28, −0.14]	−0.09* [−0.17, −0.01]	−0.24** [−0.31, −0.16]	−0.20** [−0.28, −0.12]	−0.23** [−0.31, −0.16]	−0.22** [−0.29, −0.15]
Psychological well-being composite	−0.19** [−0.27, −0.12]	−0.02 [−0.10, 0.06]	−0.21** [−0.29, −0.13]	−0.24** [−0.31, −0.17]	−0.28** [−0.35, −0.21]	−0.23** [−0.30, −0.15]
Social well-being composite	−0.14** [−0.21, −0.05]	−0.02 [−0.09, 0.06]	−0.10** [−0.17, −0.02]	−0.18** [−0.26, −0.10]	−0.24** [−0.31, −0.17]	−0.17** [−0.24, −0.09]
Self-evaluated mental/emotional health	−0.19** [−0.26, −0.11]	−0.11** [−0.19, −0.04]	−0.16** [−0.24, −0.07]	−0.19** [−0.27, −0.12]	−0.21** [−0.28, −0.14]	−0.15** [−0.22, −0.08]
Perceived control	−0.18** [−0.25, −0.10]	−0.01 [−0.10, 0.07]	−0.17** [−0.25, −0.10]	−0.20** [−0.27, −0.13]	−0.18** [−0.26, −0.10]	−0.23** [−0.31, −0.15]
	*SD*(*M*)_Personal_					
Physical health						
IADL	−0.11* [−0.20, −0.04]	−0.04 [−0.13, 0.04]	−0.08* [−0.16, −0.01]	−0.16** [−0.23, −0.09]	−0.11* [−0.19, −0.04]	−0.11* [−0.19, −0.03]
Chronic health conditions	0.09* [−0.00, 0.17]	0.02 [−0.07, 0.12]	0.07 [−0.02, 0.15]	0.10* [0.−1, 0.20]	0.04 [−0.05, 0.13]	0.08* [0.00, 0.16]
Vigorous activity	−0.07 [−0.14, 0.01]	−0.00 [−0.08, 0.08]	−0.07 [−0.15, −0.00]	−0.06 [−0.14, 0.01]	−0.11* [−0.18, −0.04]	−0.13** [−0.20, −0.05]
Moderate activity	−0.03 [−0.12, 0.05]	0.04 [−0.03, 0.12]	−0.03 [−0.11, 0.04]	−0.08* [−0.16, −0.00]	−0.06 [−0.14, 0.02]	−0.07 [−0.15, 0.02]
Self-evaluated physical health	−0.14** [−0.22, −0.06]	−0.06 [−0.13, 0.01]	−0.10* [−0.19, −0.03]	−0.15** [−0.22, −0.09]	−0.14** [−0.21, −0.07]	−0.10* [−0.16, −0.02]
Health compared to others your age	−0.09* [−0.17, −0.01]	0.02 [−0.06, 0.09]	−0.06 [−0.13, 0.02]	−0.10* [−0.17, −0.02]	−0.10* [−0.17, −0.02]	−0.03 [−0.11, 0.04]
Cognitive functioning						
BTACT composite	−0.07 [−0.14, 0.00]	−0.05 [−0.12, 0.02]	−0.02 [−0.10, 0.05]	−0.13* [−0.20, −0.06]	−0.13* [−0.20, −0.05]	−0.08* [−0.15, 0.00]
Episodic memory	−0.04 [−0.13, 0.04]	−0.01 [−0.08, 0.07]	−0.07 [−0.14, 0.00]	−0.09* [−0.16, −0.02]	−0.06 [−0.14, 0.03]	−0.06 [−0.13, 0.02]
Executive functioning	−0.06 [−0.13, 0.01]	−0.06 [−0.13, 0.02]	0.00 [−0.07, 0.08]	−0.10* [−0.17, −0.03]	−0.12* [−0.19, −0.05]	−0.07 [−0.13, 0.01]
Memory compared to others your age	−0.13** [−0.20, −0.06]	−0.03 [−0.10, 0.05]	−0.10* [−0.18, −0.02]	−0.17** [−0.24, −0.09]	−0.16** [−0.23, −0.08]	−0.11* [−0.18, −0.04]
Personality in intellectual aging contexts	−0.03 [−0.11, 0.04]	0.04 [−0.04, 0.12]	−0.03 [−0.10, 0.05]	−0.07 [−0.15, 0.00]	−0.09* [−0.16, −0.01]	−0.11* [−0.19, −0.03]
Mental well-being						
Satisfaction with life	−0.10* [−0.19, −0.02]	−0.05 [−0.13, 0.02]	−0.11* [−0.19, −0.02]	−0.07 [−0.15, 0.01]	−0.08* [−0.16, −0.00]	−0.09* [−0.18, −0.01]
Psychological well-being composite	−0.09* [−0.17, −0.01]	−0.00 [−0.08, 0.07]	−0.08* [−0.16, 0.00]	−0.10* [−0.18, −0.02]	−0.13** [−0.21, −0.05]	−0.09* [−0.17, −0.01]
Social well-being composite	−0.08* [−0.16, −0.01]	−0.00 [−0.08, 0.07]	−0.03 [−0.10, 0.05]	−0.12* [−0.19, −0.04]	−0.15** [−0.23, −0.08]	−0.11* [−0.19, −0.04]
Self-evaluated mental/emotional health	−0.11* [−0.18, −0.03]	−0.09* [−0.16, −0.02]	−0.06 [−0.14, 0.03]	−0.09* [−0.16, −0.02]	−0.10* [−0.17, −0.02]	−0.04 [−0.12, 0.04]
Perceived control	−0.09* [−0.17, −0.01]	0.01 [−0.07, 0.09]	−0.04 [−0.11, 0.04]	−0.10* [−0.17, −0.02]	−0.08* [−0.15, 0.01]	−0.12* [−0.20, −0.05]

*Notes*: *N* = 716. BTACT = Brief Test of Adult Cognition by Telephone; IADL = instrumental activities of daily living. *SD*(*M*)_Personal_ refers to the partial correlations between *SD*_Personal_ and one-time health and well-being variables, controlling for *M*_Personal_. See Supplementary Materials for descriptions of each health and well-being variable.

*p < .05. **p < .01.

#### Personal standard deviation

Table 3 also shows that *SD*_personal_ of daily successful aging was negatively correlated with 13 out of 15 physical health, cognitive functioning, and mental well-being variables (from *r* = −0.23 with self-evaluated physical health to *r* = −0.09 with BTACT). The exceptions were moderate physical activity (*r* = −0.07 [−0.16, 0.01]) and episodic memory (*r* = −0.07 [−0.14, 0.01]). That is, older adults whose daily successful aging levels varied more over the data collection period tended to score lower in physical health, cognitive functioning, and mental well-being on the one-time measures. When controlling for the *M*_personal_, the partial correlations among *SD*_personal_ and health and well-being variables weakened, while some in each category remained statistically significant (9 out of 15), including IADL, chronic health conditions, memory compared to others, satisfaction with life, current life rating, psychological well-being, social well-being, self-evaluated mental/emotional health, and perceived control over life.

## Discussion and Implications

Examining successful aging intensively within-person, our study revealed that it indeed varied in everyday life (H1), was responsive to day-to-day events and activities mostly as anticipated (H2), and was associated with most one-time health and well-being indicators showing satisfactory convergent validity (H3), with participants who scored high in daily successful aging demonstrating generally better health, functioning, and well-being as indicated by the one-time measurements.

Although successful aging showed evidence of within-person daily variation, the dimensions differed to some degree on the proportion of within-person daily variations. Nevertheless, the within-person variance was never greater than 50% of the total variance. Such variations may seem small on the surface and may partially account for the view of successful aging as a static construct. To put this in context, other intensive longitudinal studies have reported similar values for the within-person variation of health, functioning, and well-being indicators. For example, the within-person variations of everyday memory problems were found to be 23% (Rickenbach et al., 2014). Even for constructs that are typically considered highly varying across time, such as affective states, the within-person variation was also less than 50% (Mukherjee et al., 2023). As such, we posit that the within-person variation found among daily successful aging indicators is largely in line with other daily assessments of important cognitive and psychological states in late adulthood.

Although our daily measure of successful aging was responsive to day-to-day events and activities mostly as anticipated, it is noteworthy that daily physical symptoms that represented the absence of disease and disability facet appeared to yield incongruent relationships with day-to-day events compared to other successful aging indicators. This may be due to measurement-related issues, such as physical symptoms being the only negatively worded items, which may also contribute to the small correlations between physical symptoms and other successful aging indicators. On the other hand, the inconsistent findings may also reflect substantive differences, given that disease and disability can be relatively stable and less influenced by daily activities and events than other facets. Future studies incorporating a measurement burst design may help pinpoint each facet’s relative stability.

Participants’ individual average across days of daily successful aging was related to all one-time measures of health and well-being, although the effects were mostly small to medium in size. Interestingly, when examining the individual dimensions of successful aging, we found some degree of domain-specificity. For example, the disease/disability and physical functioning indicators were the only two that correlated with all one-time physical health measures. Additionally, only the cognitive functioning indicator was correlated with all one-time measures of cognitive test performances. Whereas the multidimensional daily successful aging measure indeed helps assess global health and functioning in late adulthood, the nuances of each domain can also be picked up by the corresponding subdimensions.

In our exploratory analyses, it appears that greater variation in daily successful aging was associated with poorer physical health, cognitive functioning, and mental well-being. Some of these correlations, despite the small effect sizes, remained even after accounting for personal means of daily successful aging. That is, for older adults with the same average level of daily successful aging, those who demonstrate greater (vs lesser) *instability* in daily successful aging are likely to report poorer health and well-being. This is consistent with studies showing that affective instability (i.e., variation of affective experience) is associated positively with symptomology of mental health conditions (e.g., Sultson et al., 2024). Interestingly, Rowe and Kahn (1997) themselves recognized the importance of intraindividuality “to determine the usual aging syndrome” (p. 436) among the older population. The implications of this finding are twofold. First, it highlights the additional predictive power of using daily over one-time instruments. If successful aging is operationalized as a one-time measure, the additional information associated with the within-person variability will be lost. Second, it suggests that promoting health, functioning, and well-being in late adulthood requires more than a short-term boost such as one-time interventions on social and productive engagement. It is equally important to develop long-term strategies to help older adults manage day-to-day demands and foster a stable and positive environment to sustain successful aging.

Conceptualizing successful aging as a within-person construct also has practical implications. Whereas identifying “successful agers” may help unveil individual differences in lifestyle and contributors to a healthy and positive late adulthood, extremely positive portrayals of old age are not always beneficial to older adults (e.g., Fung et al., 2015). The key appears to lie in whether older adults perceive aging as controllable and successful aging as achievable. Besides physical activities, which are consistently shown to foster successful aging (e.g., Britton et al., 2008; Depp & Jeste, 2006), our findings complement the literature by showing the effects of day-to-day stressors and positive events on daily successful aging. These events are considerably easier targets for interventions than “static” factors such as midlife socioeconomic status and personality (e.g., Britton et al., 2008; see also Serrat et al., 2024, for a review on personality and successful aging).

## Strengths, Weaknesses, and Future Directions

Our daily diary data were ideal for investigating the within- and between-person variability of successful aging and examining the day-to-day contributors to aging well. However, we acknowledge that our successful aging items are by no means a perfect representation of the construct, reflecting the constraints of performing analyses on secondary data. As we have demonstrated the possibility of operationalizing successful aging at the daily level, we recommend future scale development work to create measures that are feasible for intensive longitudinal designs such as experience sampling methods (Csikszentmihalyi & Larson, 1987). We consider such work to be important, especially at the early stage of the investigation, to also address the long-standing concerns of measurement inconsistency across successful aging studies (Katz & Calasanti, 2015).

Despite using a national sample of older adults, we are also aware of the great heterogeneity regarding health and functioning in late adulthood within and beyond the sample (e.g., Stringfellow et al., 2024). On the one hand, we anticipate that older or less healthy individuals may have greater day-to-day variability in pain perceptions and daily physical symptoms. On the other hand, the literature suggests that age is negatively associated with affect and well-being variability (Röcke & Brose, 2013), which are correlated with some successful aging components. To test these possibilities empirically, it is essential that future replications target people in the fourth age. Finally, it would be ideal for future work to incorporate alternative conceptualizations of successful aging, especially because the literature has suggested cultural differences (our study included mostly White American older adults) and discrepancies between researcher-prescribed and older adults’ self-defined features of successful aging (e.g., Phelan et al., 2004; Reich et al., 2020).

This study represents an important step toward demonstrating the possibility and utility of conceptualizing and operationalizing successful aging as a state-like (vs trait-like) construct that is susceptible to the effects of day-to-day events and activities. Overall, the findings show successful aging to be dynamic and responsive to day-to-day stressors and positive events. With this understanding, it may be useful to investigate how participating in certain activities, such as volunteering, can affect successful aging using intensive longitudinal methods. Instead of *inter*individually classifying older adults as “successful agers” or not, we could focus on *intra*individually fostering higher levels of successful aging in everyday life.

## Supplementary Material

gnae121_suppl_Supplementary_Materials

## Data Availability

Analysis syntax can be retrieved at https://osf.io/bc8hs/.
