# Supplementary material for: Capturing Successful Aging in Daily Life: Exploring the Intensive Longitudinal Findings From a U.S. National Sample
Source: Gerontologist. 2024 Sep 2;65(1):gnae121. doi: 10.1093/geront/gnae121 (PMC11638773; doi:10.1093/geront/gnae121)
Supplement: gnae121_suppl_Supplementary_Materials [file gnae121_suppl_supplementary_materials.docx]

**Supplementary Material**

**Further Descriptions of the One-Time Health and Well-being Variables**

**Physical** **Health**

Physical health was measured using six variables from the MIDUS 2 project.

***Instrumental Activities of Daily Living (IADL)***

IADL was a composite score from the scale developed by Lawton and Brody (1969). Participants were asked to answer 7 items about how much their health limits them in completing certain activities, for example, “lifting or carrying groceries” and “walking several blocks.” Items were measured on a 4-point scale ranging from 1 (*All the time*) to 4 (*Not at all*), with higher scores reflecting less limit, or higher performance, in the activities of daily life.

***Chronic Health Conditions***

Chronic health conditions were measured based on the total number of 30 chronic conditions (e.g., asthma) that participants indicated having experienced in the past 12 months.

***Self-Evaluated Physical Health***

Participants were asked to rate their physical health on a 5-point scale, ranging from 1 (*Excellent*) to 5 (*Poor*). Answers were reverse-coded, such that higher scores reflected better self-evaluated physical health.

***Health Compared to Others Your Age***

Participants were asked “compared to others your age, how would you rate your health?” They responded on a 5-point Likert scale with 1 (*Excellent*) to 5 (*Poor*). These values were then reverse-coded, with a higher score reflecting a more positive self-assessment of the participant’s health.

***Vigorous Activity***

Participants answered six survey items asking how often they engaged in vigorous physical activity, such as running or lifting heavy objects, in a variety of settings (e.g., chores, leisure, in winter or summer) with a 6-point Likert scale from 1 (*Several times a week*) to 6 (*Never*). Items were reverse-coded, with higher scores reflecting more frequent engagement in vigorous activities across settings. Cronbach’s alpha was .87.

***Moderate Activity***

Participants answered six survey items asking how often they engaged in vigorous physical activity, such as vacuuming or bowling, in a variety of settings (e.g., chores, leisure, in winter or summer) with a 6-point Likert scale from 1 (*Several times a week*) to 6 (*Never*). Items were reverse-coded, with higher scores reflecting more frequent engagement in vigorous activities across settings. Cronbach’s alpha was .85.

**Cognitive Functioning**

Cognitive functioning was measured using the following five variables taken from the MIDUS 2 and MIDUS 2 Cognitive Functioning projects.

***Brief Test of Adult Cognition (BTACT)***

The BTACT (Tun & Lachman, 2006) composite was calculated as the mean of z-scores for 6 subtests - immediate word list recall, delayed word list recall, digits backwards, category fluency, number series, and backward counting, with higher scores reflecting better cognitive functioning. The composite showed good internal consistency (α = .712).

***Episodic Memory***

The episodic memory variable was extracted from the MIDUS2 Cognitive Project data. Through telephone interviews, participants completed the episodic memory test with immediate and delayed recall of 15 words (Tun & Lachman, 2006). This variable was created as the mean of the Z-scores of immediate and delayed word list recall subtests, with higher scores representing better test performance.

***Executive Functioning***

Through telephone interviews, participants completed five executive functioning tasks (Tun & Lachman, 2006), namely digits backwards (repeating digits in reverse order), category fluency (animal naming in one minute), number series (deducing the pattern of series of numbers), and backward counting (counting backwards from 100 in 30 seconds), and stop and go switch tasks (task switching and inhibitory control). This variable was calculated as the mean of the Z-scores of the performance scores of each task, with higher scores representing better test performance.

***Memory Compared to Others Your Age***

Participants were asked “compared to others your age, how would you rate your memory?” Participants responded on a 5-point Likert scale from 1 (*Excellent*) to 5 (*Poor*). These values were then reverse-coded, with higher scores reflecting a more positive self-assessment of the participant’s memory.

***Personality in Intellectual Aging Contexts***

The Personality in Intellectual Aging Contexts (PIC; Lachman et al., 1982) scale was a composite score of nine items that assessed their beliefs about their intellectual capabilities. For example, “I don’t remember things as well as I used to.” Participants rated on a 7-point scale how strongly they agreed with the statement from 1 (*Strongly agree*) to 7 (*Strongly disagree*). The PIC composite score was created by calculating the mean of the items, with a higher score reflecting a more positive self-perception of an individual’s intellectual abilities. Cronbach's alpha was .73.

**Mental Well-Being**

Mental well-being was measured using five variables from the MIDUS 2 project.

***Life Satisfaction***

The Life Satisfaction scale (Prenda & Lachman, 2001) was composed of 6 items, each asking about a specific domain of their life - those being “life overall,” “health,” “work,” “relationship with children,” and “relationship with spouse/partner.” For example, in relation to health, participants were asked ‘how would you rate your health these days?’ Items were measured on a scale from 0 (*the worst possible*) to 10 (*the best possible*), with higher scores reflecting higher levels of life satisfaction in the domain area. Higher mean scores reflect higher levels of overall life satisfaction.

***Psychological well-being***

Psychological well-being (Ryff, 1989) was measured by 42-item scale with six dimensions, those being autonomy, environmental mastery, personal growth, positive relations with others, purpose in life, and self-acceptance. Participant rated how strongly they agreed with statements such as “Most people see me as loving and affectionate” how strongly on a 7-point scale ranging from 1 (*Strongly agree*) to 7 (*Strongly disagree*). Scores were calculated by first reverse-coding positively worded items, and then totaling the sum of the values, such that higher scores reflect greater levels of psychological well-being. Cronbach’s alpha was .93.

***Social Well-Being***

Social well-being (Keyes, 1998) was measured by a 14-item scale with five dimensions, namely meaningfulness of society, social integration, acceptance of others, social contributions, and social actualization. Participants responded to each item on a 7-point scale ranging from 1 (*Strongly agree*) to 7 (*Strongly disagree*), with higher scores reflecting a higher standing in the scale. A sample item was “I don’t feel I belong to anything I’d call a community.” Scores were calculated by first reverse-coding positively worded items, and then totaling the sum of the values, such that higher scores reflect greater levels of social well-being. Cronbach’s alpha was .82.

***Self-Evaluated Mental/emotional Health***

As a single item, participants were asked to rate their current mental/emotional health on a 5-point scale, ranging from 1 (*Excellent*) to 5 (*Poor*). This was reverse-coded so that higher scores indicated more positively self-evaluated mental/emotional states.

***Perceived Control***

Participants responded to a 12-item sense of control scale (Lachman & Weaver, 1998) with statements such as “I can do just about anything I really set mind to” on a 7-point scale from 1 (*Strongly agree*) to 7 (*Strongly disagree*). The composite was calculated by first reverse-coding positively worded items then taking the mean of all items. Higher scores indicated greater overall perceived control. Cronbach’s alpha was .87.

Supplementary References

Diener, E., Emmons, R., Larsen, R. J., & Griffin, S. (1985). The satisfaction with life scale. *Journal of Personality Assessment, 49*(1), 71–75. https://doi.org/10.1207/s15327752jpa4901_13

Keyes, C. L. M. (1998). Social well-being. *Social Psychology Quarterly, 61*, 121–140. https://doi.org/10.2307/2787065

Lachman, M. E., Baltes, P., Nesselroade, J. R., & Willis, S. L. (1982). Examination of personality-ability relationships in the elderly: The role of the contextual (interface) assessment mode. *Journal of Research in Personality, 16*(4), 485–501. https://doi.org/10.1016/0092-6566(82)90007-1

Lachman, M. E., & Weaver, S. L. (1998). The sense of control as a moderator of social class differences in health and well-being. *Journal of Personality and Social Psychology, 74*(3), 763–773. https://doi.org/10.1037/0022-3514.74.3.763

Lawton, M. P., & Brody, E. M. (1969). Assessment of older people: Self-maintaining and instrumental activities of daily living. *The Gerontologist, 9*, 179–186. https://doi.org/10.1093/geront/9.3_Part_1.179

Prenda, K. M., & Lachman, M. E. (2001). Planning for the future: a life management strategy for increasing control and life satisfaction in adulthood. *Psychology and Aging, 16*(2), 206–216. https://doi.org/10.1037/0882-7974.16.2.206

Ryff, C. (1989). Happiness is everything, or is it? Explorations on the meaning of psychological well-being. *Journal of Personality and Social Psychology, 57*, 1069–1081. https://doi.org/10.1037/0022-3514.57.6.1069

Tun, P. A., & Lachman, M. E. (2006). Telephone assessment of cognitive function in adulthood: the Brief Test of Adult Cognition by Telephone. *Age and Ageing, 35*(6), 629–632. https://doi.org/10.1093/ageing/afl095

Watson, D., Clark, L. A., & Tellegen, A. (1988). Development and validation of brief measures of positive and negative affect: The PANAS scales. *Journal of Personality and Social Psychology*, *54*(6), 1063–1070. https://doi.org/10.1037/0022-3514.54.6.1063
